# Correlation between ER, PR, HER-2, and Ki-67 with the risk of bone metastases detected by bone scintigraphy in breast cancer patients: A cross sectional study

**DOI:** 10.1016/j.amsu.2021.102532

**Published:** 2021-06-29

**Authors:** Hanif Afkari, Firdian Makrufardi, Basuki Hidayat, Hendra Budiawan, Achmad Hussein Sundawa Kartamihardja

**Affiliations:** a Nuclear Medicine and Molecular Imaging Division, Department of Radiology, Faculty of Medicine, Public Health and Nursing, Universitas Gadjah Mada/Dr. Sardjito General Hospital, Yogyakarta, Indonesia; b Department of Nuclear Medicine and Molecular Imaging, Universitas Padjajaran/Dr. Hasan Sadikin General Hospital, Bandung, Indonesia

**Keywords:** Ki-67, Bone metastases, Bone scintigraphy

## Abstract

**Background:**

Breast cancer is one of the most common cancers in women. About 30%–85% of breast cancers will metastasize to the bone during the course of the illness. Many studies have shown that molecular marker/subtypes can be useful in determining incidence of different and inconsistent bone metastases. This study aimed to determine the correlation of the risk of bone metastases in breast cancer based on the expression of molecular markers.

**Methods:**

The research was conducted retrospectively by searching patients' medical record data. The target population of this study was all patients diagnosed with breast cancer who came to our tertiary hospital in the Nuclear Medicine and Molecular Imaging Department from January 2012 to December 2016.

**Results:**

One hundred and thirty patients (n = 130) were enrolled during the study period with characteristics of sex, age, and immunohistochemical/molecular subtype examination that underwent bone scintigraphy. Mean of age was 50.2 (23–79) years. There were no significant correlations between ER, PR, and HER-2 expressions with bone metastases in breast cancer patients. Ki-67 was showed to be correlated with bone metastases in breast cancer patients in our bivariate analysis. Molecular subtype/markers had no statistically significant correlation with bone metastases in patients with breast cancer.

**Conclusion:**

Ki-67 with high proliferation index was the most powerful molecular marker to determine the risk of bone metastases. The prevalence of bone metastases in the group with Ki-67 expression with high proliferation (≥20) was 1.8 times greater than the prevalence of bone metastases in the weakest HER-2 group.

## Highlights

• Molecular marker can be useful in determining bone metastasis.
• Ki-67 can determine the risk of bone metastasis.
• No significant correlations between ER, PR, and HER-2 expressions with bone metastasis.

## 1 Introduction

Breast cancer is one of the most common cancers in women. According to GLOBOCAN/IARC in 2012, it accounted for about 1.67 million cases and 25% of all cancers. The World Health Organization (WHO) data in 2012 showed the prevalence of breast cancer in Southeast Asia was as much as 2.04 million cases, while the incidence of breast cancer in Indonesia was 40 per 100,000 population [1,2].

Breast cancer has a range of presentations with different molecular subtypes, with different biomolecular, pathological, and genetic features with different clinical and therapeutic response results, thus breast cancer is termed as a heterogeneous disease. These molecular markers are known to be closely related to oncogenic transformation, cancer cell proliferation, tumor growth, treatment choice, and prognosis of breast cancer [3]. In addition, some previous studies have also found that the expression of molecular markers of immunohistochemical examination results may be related to the incidence of metastases in breast cancer [4,5]. Several studies have suggested that the molecular subtypes of breast cancer are associated with their unique pattern of metastases. Grouping of molecular markers into different molecular subtypes will provide information on prognosis and therapeutic responses [6].

Bone is the most common metastatic location of breast cancer. About 30%–85% of breast cancers will metastasize to the bone during the course of the illness. Bone scintigraphy with 99 mTc-methoxyethylenephosphonate has high sensitivity, but low specificity. Bone scintigraphy is not routinely performed in patients with breast cancer but is usually used if there are complaints of bone pain. The National Comprehensive Cancer Network (NCCN) in 2016 recommended that bone scintigraphy should be done from stage IIIA to stage IV, whereas typically it is in stage I-IIB when there are complaints of bone pain or increased alkaline phosphatase. Early identification of bone metastases will change the pattern of breast cancer management [7,8].

The molecular subtype marker in breast cancer is associated with a unique pattern of metastases. Many studies have shown that molecular subtypes can be used in determining the incidence of different and inconsistent bone metastases. It is possible that the occurrence of bone metastases in breast cancer can be determined by molecular markers which are a component in the molecular subtype [8]. If the results of a molecular marker examination indicate the presence of suspected bone metastases, bone scintigraphy can be performed without waiting for bone pain or advanced stage [9]. This study was aimed to determine the correlation of the risk of bone metastases in breast cancer based on the expressions of molecular markers: estrogen receptor (ER), progesterone receptor (PR), human epidermal growth factor receptor-2 (HER-2) and Ki-67.

## 2 Material and methods

### 2.1 Patients and outcomes

The research was conducted retrospectively through the search of patients' medical record data. This cross-sectional study included 130 participants, consisting of all female patients. The target population of this study was all patients who had been diagnosed with breast cancer who came to our tertiary hospital in the Nuclear Medicine Department and Molecular Imaging from January 2012 to December 2016 and met the inclusion criteria. The inclusion criteria in this study were: 1) have medical record data showing the results of breast cancer by pathologic anatomic result, 2) have the results of molecular markers ER, PR, HER-2 and Ki-67, and 3) have bone scintigraphy results with osteoblastic bone metastases.

The diagnosis of breast cancer in our institution was established using clinical manifestation, imaging and biopsy examination [2]. We determined histology and subtype based on results in patients who had undergone surgery for the first time and had not given any therapy. Patients that have more than one primary tumor and have been receiving treatment with therapeutic effects that can affect bone sculpting results were excluded from this study. This study has been reported in line with the Strengthening the Reporting of Cohort Studies in Surgery (STROCSS) criteria [25].

Patients with complaints of bone pain and suspicion of bone metastases by an oncologist will undergo a bone scintigraphy at our institution. The presence or absence of bone metastases were indicated by the appearance of hot-spots/multiple hot-spots which supports the diagnosis of bone metastases. The metastatic assessment that used was a hallmark of osteoblast metastases: single lesion/multiple lesions, flare phenomenon, elongation, and doughnut sign [26]. However, patients with symmetrical uptake in joints, location of bone pain that was confirmed not because of a trauma, degenerative process and normal distribution of radiopharmaceuticals were excluded from diagnosis of bone metastases.

### 2.2 Statistical analysis

Numerical data are presented with mean and standard deviation (SD) or median and range, while categorical data are presented with frequency and percentage. The molecular marker correlation/molecular subtypes of breast cancer with bone metastases were analyzed by chi-squared tests, while the epidemiological measures used prevalence and 95% confidence interval (CI). The IBM SPSS Statistics 23rd version software (IBM Corp., Chicago) was used to analyze data. All statistical tests with *p* < 0.05 were considered as significant.

## 3 Results

One hundred and thirty patients (n = 130) were enrolled during the study period with characteristics of sex, age, and immunohistochemical/molecular subtype examination that underwent bone scintigraphy. The subjects’ characteristics (Table 1) diagnosed with breast cancer were all female (100%) with mean of age of 50.2 (23–79) years. The molecular markers of ER, PR and HER-2 were divided to positive and negative (63%; 37%), (57%; 43%), and (33%; 67%), respectively. Meanwhile, the subject data with complete Ki-67 included 91 subjects with the majority of high proliferation (71%).

**Table 1:** Subjects’ characteristics.

Characteristics	n	%
Sex		
Male	0	0
Female	130	100
Age		
<50 year	59	45
≥50 year Mean (min-max)	71 50.5 (23–79)	55
IHC examination ER Positive Negative	82 48	63 37
PR Positive Negative HER2 Positive Negative KI-67 (n = 91) Low proliferation High proliferation	74 56 43 87 26 65	57 43 33 67 29 71
Molecular subtype (n = 114) Luminal A Luminal B HER2-*enriched* TNBC Bone metastases Positive Negative	17 59 20 18 70 60	13 45 15 14 54 46

ER: estrogen receptor, PR: progesterone receptor, HER-2: human epidermal growth factor receptor-2, TNBC: triple-negative breast cancer.

The total number of subjects (n = 130) with bone scintigraphy results showed that the presence of positive bone metastases was 54%, while bone scintigraphy results showed 46% with no bone metastases.

Molecular subtype markers were divided into four molecular subtypes (Table 2). Complete subject data were grouped as 114 subjects with Luminal A 13% (17/114), Luminal B 45% (59/114), HER2-enriched 15% (20/114) and triple-negative breast cancer (TNBC) 14% (18/114).

**Table 2:** Immunohistochemical criteria to determine molecular subtype.

Subtype	ER	PR	HER2	KI-67
Luminal A	Positive ER and/or positive PR		Negative	Low
Luminal B	Positive ER and/or positive PR		Negative	High
	Positive ER and/or positive PR		Positive	
HER2-*enriched*	Negative	Negative	Positive	
TNBC	Negative	Negative	Negative	

ER: estrogen receptor, PR: progesterone receptor, HER-2: human epidermal growth factor receptor-2, TNBC: triple-negative breast cancer.

In the bivariate analysis (Table 3), in each molecular marker; ER, PR, and HER-2 in bone metastases incidence/relative prevalence (RP) with confidence interval (95% CI) and *p* (chi-squared test) were 1.05 (0.75–1.47) with *p* = 0.758; 0.90 (0.65–1.23) with *p* = 0.512 and 0.99 (0.71–1.39) with *p* = 0.954. The high proliferation of Ki-67 molecular markers on bone metastases incidence with prevalence ratio = 1.80, 95% CI (0.97–3.33) and *p* = 0.034. There were no significant correlations between ER expression, PR, HER-2 with bone metastases in breast cancer patients. Ki-67 was correlated with bone metastases in patients with breast cancer.

**Table 3:** Bivariate analysis of breast cancer subtypes.

IHC	Bone metastases	Total PR (95% CI)	*p*-value	
	**Yes**	**No**		
				
**ER**				
Positive	45 (55)	37 (45)	74 (100) 1.05 (0.75–1.47)	0.758
Negative	25 (52)	23 (48)	48 (100)	
PR				
Positive	38 (51)	36 (49)	74 (100) 0.90 (0.65–1.23)	0.512
Negative	32 (57)	24 (43)	56 (100)	
HER-2				
Positive	23 (54)	20 (46)	43 (100) 0.99 (0.71–1.39)	0.954
Negative	47 (54)	40 (46)	87 (100)	
Ki-67				
Strong (≥20%)	36 (55)	29 (45)	65 (100) 1.80 (0.97–3.33)	0.034
Weak (<20%)	8 (31)	18 (69)	26 (100)	

ER: estrogen receptor, PR: progesterone receptor, HER-2: human epidermal growth factor receptor, CI: Confidence interval, RP: Relative prevalence.

In multivariate analysis (Table 4), molecular markers were grouped in the four molecular sub-types based on the incidence of bone metastases with TNBC as reference, with relative prevalence (RP) and 95% CI for HER-2 enriched, Luminal B and Luminal A: 0.90 (0.49, 1.64), 1.10 (0.69, 1.74) and 0.53 (0.23; 1.23) with *p* values: 0.732; 0.679; and 0.118, respectively There was no statistically significant correlation between subtype and bone metastases in patients with breast cancer.

**Table 4:** Multivariate analysis of breast cancer subtypes.

	Bone metastases				
Subtype	Yes	No	Total	PR (95% CI)	*P*-value
TNBC	10 (56)	8 (44)	18 (100)	Ref	
HER2-enriched	10 (50)	10 (50)	20 (100)	0.90 (0.49–1.64)	0.732
Luminal B	36 (61)	23 (39)	59 (100)	1.10 (0.69–1.74)	0.679
					
Luminal A	5 (29)	12 (71)	17 (100)	0.53 (0.23–1.23)	0.118

HER-2: human epidermal growth factor receptor-2, TNBC: triple-negative breast cancer.

RP: Relative prevalence, Ref: Reference.

## 4 Discussion

The molecular markers of ER, PR, HER-2 and Ki-67 are routinely performed in breast cancer patients both during screening and diagnosis. Single molecular markers, including ER, PR, HER2, and Ki-67 as disease proliferation signs have been used for several years to predict breast cancer prognosis and therapeutic guidance [8,9]. This study aimed to explain which molecular markers are most influential to the risk of bone metastases prevalence in patients with breast cancer who have never been treated since the diagnosis with bone scintigraphy.

Ki-67 expression is usually predicted as a positive percentage of tumor cells in staining with antibodies, with core staining being the most common criterion of the proliferation index. Ki-67 Index will express cells that proliferate in phases G1, S, G2, and M except the G0 phase of the cell cycle. Low Ki-67 levels occur in the G1 and S phases and increase to peak-level during mitosis. Ki-67 acts as a predictive factor, and the Ki-67 proliferation index is a molecular marker used to assess the activity of cell proliferation that is often used in detecting breast cancer [8]. Ki-67 is very important to demonstrate cell proliferation, because downregulation of Ki-67 uses antisense nucleotides that prevent proliferation. Ki-67 is strictly controlled and regulated, which illustrates an important role in cell proliferation. Bridger et al. described the role of Ki-67 in organizing DNA, based on its localization outside the nucleolus during the early G1 cycle, along with the centrometer and DNA satellites. Ki-67 is also known to be associated with DNA. Ki-67 is a protein that is expressed in the cell nucleus during the cell cycle. Generally, the higher the Ki-67 expression, the more proliferation of tumors [10].

Many breast cancer studies showed clearly, that there are statistically significant correlations with clinical outcomes, both in univariate and multivariate analysis. Strong relationships have been noted between the positive percentage of Ki-67 cells with core grading, age, and mitosis rates. Uncontrolled proliferation is one of the main cancer characteristics, since proliferation is one of the major factors associated with prognosis [9]. Several studies have shown that breast cancer with Ki-67 expression more than 20–50% has a high risk of metastases, indicating that there are statistically significant correlations with clinical outcomes, such as disease-free survival and overall survival [10,11].

The high levels of Ki-67 in breast cancer as a molecular marker involved in cell proliferation are associated with poor outcomes [12]. A study reported there was a significant increase in Ki-67 between primary breast cancer and breast cancer with metastases [11]. Another study found that prognostic factors (ER, PR, and HER-2) with a higher Ki-67 index had a worse prognosis [13].

This study also found that there was no significant relationship between ER expression, PR, HER-2 with bone metastases in patients with breast cancer. The prevalence of bone metastases in groups with positive ER, PR and HER-2 expression were similar to the negative ER, PR, and HER-2 expression groups. Previous study explained that positive ER expression was relatively high relative to increased risk of bone metastases. The PR expression was almost the same, but the statistical difference was not significant. The association of positive PR expression data with metastases was inconsistent. One study found no significant effect between ER and PR expression on the possibility of bone metastases [14]. Some previous retrospective studies reported varying levels of mismatch between the expressions of ER molecules, PR and HER-2 primary tumors with metastases. In this report, the level of nonconformity for hormonal receptor status expression (ER/PR) ranged from 18% to 54%, while HER-2 expression ranged from 0% to 34% [15]. Another study found different results in ER, PR and HER-2 primary and metastatic tumors with prevalences that were 19.1%, 11.8% and 14.8%, respectively [18].

This incompatibility in results may be caused by some of the heterogeneity factors of the underlying disease. The heterogeneity in the expression of the genes will develop in the population of the tumor cells, for example, as a result of the effects of genomic instability and the accumulation of various mutations and other genetic deviations. The biologically subdivided clonal subspecies of certain tumors can develop independently from most cancerous cells and cause metastases that are genetically distinct from the majority of cells in primary tumors. Due to genetic heterogeneity, differences in metastatic sites present in biological disorders as well as in primary tumors [[16], [17], [18]].

Molecular markers are often grouped into other molecular sub-types. The main purpose of this grouping is to determine that there is a subtype of breast cancer with different gene expression patterns and different prognoses [19]. Further studies have found a difference in prognosis and chemotherapy responses associated with the molecular sub-type of a particular patient cohort. This observation reinforces the hypothesis that the pattern of expression of a specific molecule gene for a particular sub-type has clinical relevance [20].

Our multivariate analysis was found no correlation between molecular subtype and bone metastases in breast cancer patients. This result can be caused by the small sample size in the molecular subtype grouping, as well as the confounding variables. Previous studies of molecular sub-type clustering were performed generally to determine recurrence, prognosis and therapeutic responses. The results primarily determined that recurrence is heavily influenced by other risk factors. Recurrence risk factors are influenced by age, early stages, tumor biological backgrounds, tumor size, lymph node involvement, histological grade, lymphovascular invasion, hormone receptor, HER-2 and menopause status [10].

Age as a risk factor for bone marrow metastases is still widely debated and the findings are contradictory, showing histological types of breast cancer also exhibit different metastatic patterns [21,22]. Menopause status may also be associated with the risk of bone metastases because estrogen is an important regulator of bone remodeling, potentially contributing to bone metastases in the bone microenvironment. Grade of cancer is related to bone metastases at low histology grade in primary breast cancer compared with cancer progression that is already differentiated [23]. The size of the tumor has been associated with bone metastases. Lymph node involvement is also an important risk factor for bone metastases. Additionally, tumor staging is considered as an independent risk factor for bone metastases [15,24].

There were some aspects of our research that needed to be considered. Because the majority of our patients were referral cases with no data in their medical records, we did not consider stages at diagnosis as a variable in our study. While some clinical and pathological variables such as axillary lymph node metastases, menopausal status, tumor size, serum concentrations of CA125 > 21.99 u/ml, CA153 > 25.42 u/ml, ALP >100.5 u/l and hemoglobin <49 g/l to be a risk factor for metastases in breast cancer [27,28], however, our study did not determine those factors with the risk of bone metastases, becoming one of our study's limitation. It should be noted that the population of this study was breast cancer patients with bone scintigraphy examination that might not represent the whole population of breast cancer patients.

Bone scintigraphy can be the one of modalities to determine bone metastases in breast cancer patients. Moreover, we suggest clinician to choose the appropriate modalities to determine bone metastases in breast cancer patients based on availability of modalities in institution, accuracy, and clinical condition of patients.

## 5 Conclusions

Ki-67 with high proliferation index was identified to be the most powerful molecular marker to determine the risk of bone metastases. The prevalence of bone metastases in the group with Ki-67 expression with high proliferation (≥20) was 1.8 times greater than the prevalence of bone metastases in the weakest HER-2 group.

## Ethical approval

This study has been approved by the Ethical Committee of Faculty of Medicine Universitas Padjajaran/Dr. Hasan Sadikin General Hospital.

## Sources of funding

The authors declare that this study had no funding source.

## Author contribution

Hanif Afkari conceived the study and critically revised the manuscript for important intellectual content. Firdian Makrufardi, Basuki Hidayat, Hendra Budiawan, and Achmad Hussein Sundawa Kartamihardja drafted the manuscript and critically revised the manuscript for important intellectual content. All authors read and approved the final draft. All authors facilitated all project-related tasks.

## Research Registration number

NCT00582920.

## Guarantor

Hanif Afkari.

## Consent

Written informed consent was obtained from the patients before joining the study. A copy of the written consent is available for review by the Editor-in-Chief of this journal on request.

### Provenance and peer review

Not commissioned, externally peer reviewed.

## Declaration of competing interest

No potential conflict of interest relevant to this article was reported.

## Registration of research studies

NCT00582920.
